# The Effect of the Oral Contraceptive Pill on Acute Glycaemic Response to an Oral Glucose Bolus in Healthy Young Women: A Randomised Crossover Study

**DOI:** 10.3390/nu16203490

**Published:** 2024-10-15

**Authors:** Julia M. E. Cree, Niamh M. Brennan, Sally D. Poppitt, Jennifer L. Miles-Chan

**Affiliations:** 1 Human Nutrition Unit, School of Biological Sciences, University of Auckland, Auckland 1010, New Zealands.poppitt@auckland.ac.nz (S.D.P.); 2 High Value Nutrition, National Science Challenge, Auckland 1023, New Zealand; 3 Riddet Institute, Massey University, Palmerston North 4442, New Zealand

**Keywords:** combined oral contraceptive, carbohydrate metabolism, postprandial glycaemia, glucose homeostasis, insulin, women’s health

## Abstract

**Background/Objective:** The oral contraceptive pill (OCP) is widely used by women worldwide, yet the influence of the OCP on carbohydrate metabolism remains under-investigated, with existing studies being few and largely cross-sectional. The study objective was to assess, for the first time, the effect of the combined OCP on postprandial glycaemic response to an oral glucose bolus, using a randomised crossover design. **Methods:** The effect of a combined monophasic OCP phase on glucose homeostasis and metabolic profile was investigated in 21 healthy young women, who were regular users of either androgenic or anti-androgenic OCP formulations. Plasma glycaemic markers (glucose, insulin and C-peptide) were assessed prior to a 60 g glucose drink (fasting) and for a further 4 h postprandially; once during the “active” (hormone-containing) pill phase and once during the “inactive” (hormone-free) pill phase of the OCP usage cycle. **Results:** Despite no change in fasting values, in androgenic pill users, postprandial glucose and insulin responses to an oral glucose bolus were ~100% and ~50% greater, respectively, during the active versus inactive phase. In contrast, in anti-androgenic pill users there was no significant change in response between the two OCP usage cycle phases. **Conclusions:** These findings highlight an acute, but potentially detrimental, influence of the combined OCP on glucose homeostasis, particularly in users of formulations containing androgenic progestogens. Given the high global prevalence of OCP use and increasingly common prolonged active pill regimens, which may continue for months, years or even decades, potential cumulative effects of such changes on metabolic risk demand further investigation.

## 1. Introduction

Metabolic syndrome (MetS) comprises dyslipidemia, hypertension, impaired glucose metabolism and abdominal obesity [1]; a constellation of risk factors for cardiometabolic diseases which have themselves been described as a “global epidemic” [2]. There is growing evidence that the incidence of MetS in women, particularly those of child-bearing age, is increasing disproportionately to that in men [1] and is contributing to increasing rates of cardiometabolic diseases [3]. For example, a comparison of NHANES data from the periods 1988 to 1994 and 2007 to 2012 shows the prevalence of MetS in women increased from 25.0% to 34.9% between these two time periods, to exceed that of men (33.4%) during the latter [4]. Furthermore, a 2008 in-depth analysis of NHANES statistics focusing on women of child-bearing age (18–44 y) estimated that 50% of the sample population either had MetS or were at increased risk of developing the condition [5]; with women of diabetic mothers almost twice as likely to exhibit a MetS phenotype than women of non-diabetic mothers [5]. Taken together, and with the notion of epigenetic inheritance now well-accepted [6,7], this has on-going significant implications for the health of future generations. However, the metabolic basis for this disparity in disease risk between the sexes is unknown.

Since its approval for contraceptive use >60 y ago, “The Pill” has become one of the most widely employed methods to prevent pregnancy, and its popularity continues to grow, with the estimated number of women using hormone-containing pills increasing from 97 to 151 million in the period from 1994 to 2019 [8]. Yet surprisingly, regardless of its extensive use, the impact of exogenous hormones in the form of the oral contraceptive pill (OCP) on metabolism, and its potentially adverse metabolic effects, have not been fully explored.

The current literature relating to the metabolic effects of OCP is often contradictory or inconclusive. Women taking oral contraception have been shown to have a 40% reduction in insulin sensitivity [9]. Yet a 2019 Cochrane review concluded “current evidence suggests no major differences in carbohydrate handling between different hormonal contraceptives in women without diabetes” [10]. However, the vast majority of this research has measured metabolic changes in non-users who were assigned OCPs for the duration of the study, with variation in the time period for assessing such changes, which may account for conflicting results. Surprisingly, there is also an absence of published research investigating differences in glucose handling and biomarkers of cardiometabolic risk between the “active” (hormone-containing) and “inactive” (hormone-free) pill-taking phases of the OCP usage cycle in established users, with the majority of studies to-date being cross-sectional or longitudinal in design. This is true despite the traditional contraceptive pill format (comprising 21 hormone-containing pills followed by 7 “inactive” pills within a 28-day cycle) providing an ideal way to assess acute, intra-individual pill-induced changes. Therefore, in light of this knowledge gap, the aim of the current study was to assess the effect of exogenous female sex hormones (as supplied by the OCP) on fasting blood glucose and, secondly, on postprandial glycaemic response to an oral glucose bolus, using a crossover study design in habitual OCP users.

## 2. Materials and Methods

### 2.1. Study Participants

Healthy young women (aged 18–40 y, with a body mass index, BMI, of 18.5–26.9 kg/m^2^) taking a combined monophasic OCP according to the manufacturer’s instructions for >3 months were recruited. Web-based software was used [11] for an a priori power analysis calculation, with a sample size (n) of 16 being determined as necessary to detect a clinically and statistically significant change (0.5 mmol/L) in fasting blood glucose with within-participant variance (standard deviation, SD) of 0.4 mmol/L [12]. Initially, 22 women were enrolled to allow for possible participant dropouts, and of these, 18 participants completed both study visits. Preliminary analysis of the results (of the initial 18 participants who completed both study visits) indicated differences in glucose homeostasis due to pill type, with pills classified as either androgenic or anti-androgenic based on the characteristics of the progestin component [13]. An additional 3 participants taking anti-androgenic pills were therefore recruited to ensure both pill-type subgroups contained a minimum of n = 8 to enable statistical comparisons between pill type. Exclusion criteria were as follows: variance in self-reported body weight greater than ±5% during the preceding 6 months, absence of regular withdrawal bleeds during the inactive phase of pill taking, a previous or current chronic medical condition such as diabetes, heart disease, cancer, gout, hypertension, endocrine or hormone-related disorder, gastrointestinal disease or immune condition, taking medication other than the OCP which could affect metabolic rate and/or glucose handling, smoking/vaping, alcohol consumption in excess of New Zealand’s Ministry of Health guidelines [14].

Due to secondary outcomes of the study related to appetite and energy metabolism (specifically glucose-induced thermogenesis) vegetarians or vegans, and those prone to claustrophobia, were also excluded. This study complied with the Declaration of Helsinki, was approved by Northern A Health and Disability Ethics Committee (ref. 18/NTA/61) and prospectively registered on the Australian/New Zealand Clinical Trial Registry (ACTRN12618000858291). All participants gave written consent.

### 2.2. Experimental Design and Study Visits

This study was a single-centre, crossover intervention. After completing a pre-screening questionnaire, potentially eligible participants attended a screening visit to provide informed consent, complete a lifestyle/diet questionnaire, and anthropometry measurements including body composition analysis by DXA (model iDXA, software version 15, GE-Lunar, Madison, WI, USA). Participants were then provided a standardised evening meal and snacks (total energy 4.0 MJ, 35 g protein, 32 g fat, 126 g carbohydrate with an overall fat-to-carbohydrate energy ratio varying between 0.52 and 0.53 depending on the combination of meal and snacks eaten) and instructed to consume this *ad libitum* by 8 pm the night before their laboratory visit, in order to control macronutrient intake prior to each test and comply with 12 h of fasting. Participants were asked to abstain from caffeine, alcohol and intense/excessive physical activity during the 24 h period prior to their visits.

On experimental days, participants arrived, having fasted, at the laboratory (8 am). Testing was conducted once during the last 7 days of the 21-day active pill phase and once during the last 3 days at the end of the inactive pill phase, with the order of these visits being randomised using a simple, single-block randomisation plan (http://www.randomizer.org; accessed 2 April 2018). Upon arrival, the participant was asked to void their bladder prior to being weighed; then they were seated whilst a peripheral venous cannula was inserted into the antecubital vein, and a fasted blood sample was collected.

Participants were seated in a clinical dialysis chair with leg support and permitted to watch a calm movie or documentary to ensure that they remained alert but relaxed during proceedings. After a seated rest of at least 30 min, participants consumed a drink comprising 60 g of glucose dissolved in 294 mL of water, flavoured with 6 mL lemon juice to improve palatability. Whilst this measure is less than the 75 g of glucose often ingested during an oral glucose tolerance test, it has been shown to sufficiently increase insulin, without nausea and other gastrointestinal disturbances often associated with the ingestion of a large glucose bolus, and allows full capture of the postprandial glucose and insulin response and glucose-induced thermogenesis (a secondary outcome of the study) within a 4 h monitoring period [15]. Participants were asked to consume the drink within 5 min; then they were monitored for a further 4 h, with a 20 min “comfort break” commencing after 2 h. The break was mandatory, and all participants were instructed to walk slowly the short distance (~5 m) to the bathroom.

In addition to the fasting (t = 0) sample, a total of 7 postprandial blood samples were collected after the glucose drink: t = 15, 30, 60, 90, 120, 180 and 240 min. Venous blood samples were immediately transferred to vacutainers (BD, Franklin Lakes, NJ, USA) containing potassium oxalate, sodium fluoride for glucose measurement or lithium heparin for lipid and hormone analyses; then, they were refrigerated for 30–60 min prior to centrifugation at 3500 RPM for 15 min at 4 °C. Resultant plasma was aliquoted and frozen at −80 °C until further analysis.

### 2.3. Plasma Analyses

Plasma samples were batch-analysed using a COBAS e411 autoanalyser (Hitachi High Technologies Corporation, Tokyo, Japan) to measure insulin, cortisol, estradiol, progester-one, testosterone, C-peptide and sex hormone binding globulin (SHBG) by electrochemi-luminescence immunoassay, and a COBAS e311 autoanalyser (Hitachi High Technologies Corporation) was used to measure plasma lipids by enzymatic colorimetric assay and glucose by enzymatic UV assay. Analyses were run using the manufacturer’s recommen-ded, commercially available reagents (Roche diagnostics, Mannheim, Germany). Samples were processed in singlicate.

### 2.4. Data Analyses

HOMA1-IR was calculated as HOMA1-IR = [fasting plasma insulin (µIU/L) × fasted plasma glucose (mmol/L)]/22.5 [16]. HOMA2-1R was calculated from glucose and C-peptide levels using the online HOMA2 calculator (version 2.2.3; Diabetes Trials Unit, University of Oxford, UK) [17]. Statistical analyses were performed using GraphPad Prism 8.4.3 (GraphPad Software Inc., La Jolla, CA, USA). Participant characteristics were compared using unpaired *t*-tests. Differences in baseline plasma parameters were assessed using two-way, repeated-measures ANOVA (pill phase and pill type) followed by Bonferroni *post hoc* testing. Timecourse data of postprandial changes (relative to fasted baseline) were assessed using a linear mixed model (LMM) to analyse repeated measures data, with time (mins), pill type (androgenic and anti-androgenic) and pill phase (active and inactive) included as fixed effects and participants treated as random effects. Differences between pill type and phase at specific timepoints were investigated using Bonferroni *post hoc* testing. Net incremental AUC (iAUC) of plasma glucose, insulin and C-peptide was calculated using the trapezoid method, including both positive and negative peaks. The level of significance was set at *p* < 0.05. Data are represented as means ± standard error of the mean (SEM).

## 3. Results

### 3.1. Participant Characteristics

In total, 25 participants were randomised into this study, with 21 completing the 2 experimental testing visits, 14/21 participants in an active–inactive sequence, and 7/21 in an inactive–active sequence. Four participants withdrew between the screening and their first laboratory visit without giving a reason. The breakdown of participant OCP usage by brand name and hormonal composition is presented in Table 1. All OCPs contained ethinylestradiol (EE); however, the progestin component, and therefore androgenic characteristics [13], differed. Sixteen participants (76%) had been using their current OCP for more than a year, three participants for 6–12 months, and two participants for between 3 and 6 months.

Age, anthropometric and body composition data are provided in Table 2. There were no significant differences in these parameters between women using pills containing an androgenic progestin and an anti-androgenic progestin.

### 3.2. Fasting (Baseline) Levels of Hormones and Biomarkers of Metabolism

Fasting (baseline) levels of hormones and biomarkers of metabolism are shown in Table 3.

### 3.3. Postprandial Glycaemic Response

**Figure 1:**
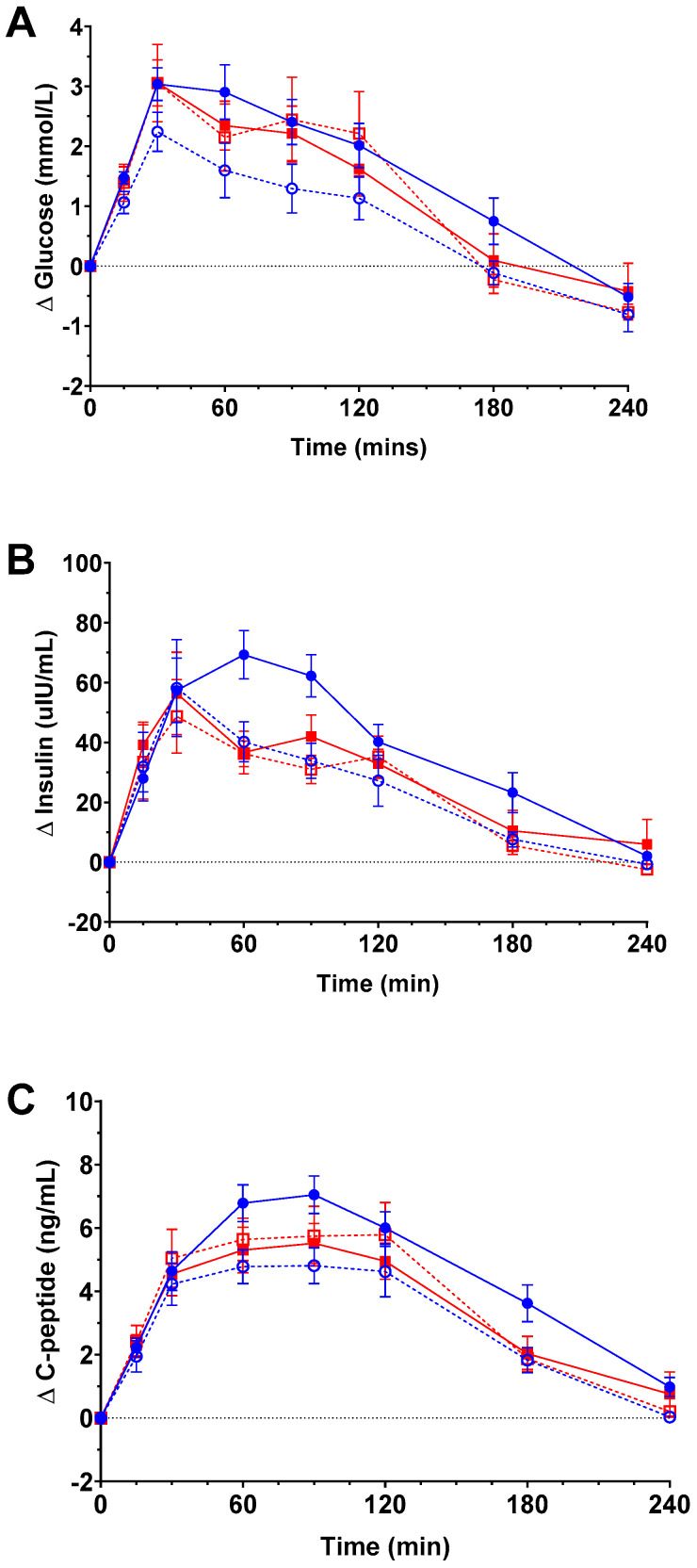
Change in plasma glucose (**A**), insulin (**B**) and C-peptide (**C**) from fasting baseline over 240 min following consumption of a 60g glucose bolus. Women using oral contraceptive pills (OCPs) containing androgenic progestogens (n = 13) during the active (blue closed circles) and inactive phases (blue open circles). Women using OCPs with anti-androgenic progestogens (n = 8) during the active (red closed squares) and inactive phases (red open squares). Mean ± SEM.

**Figure 2:**
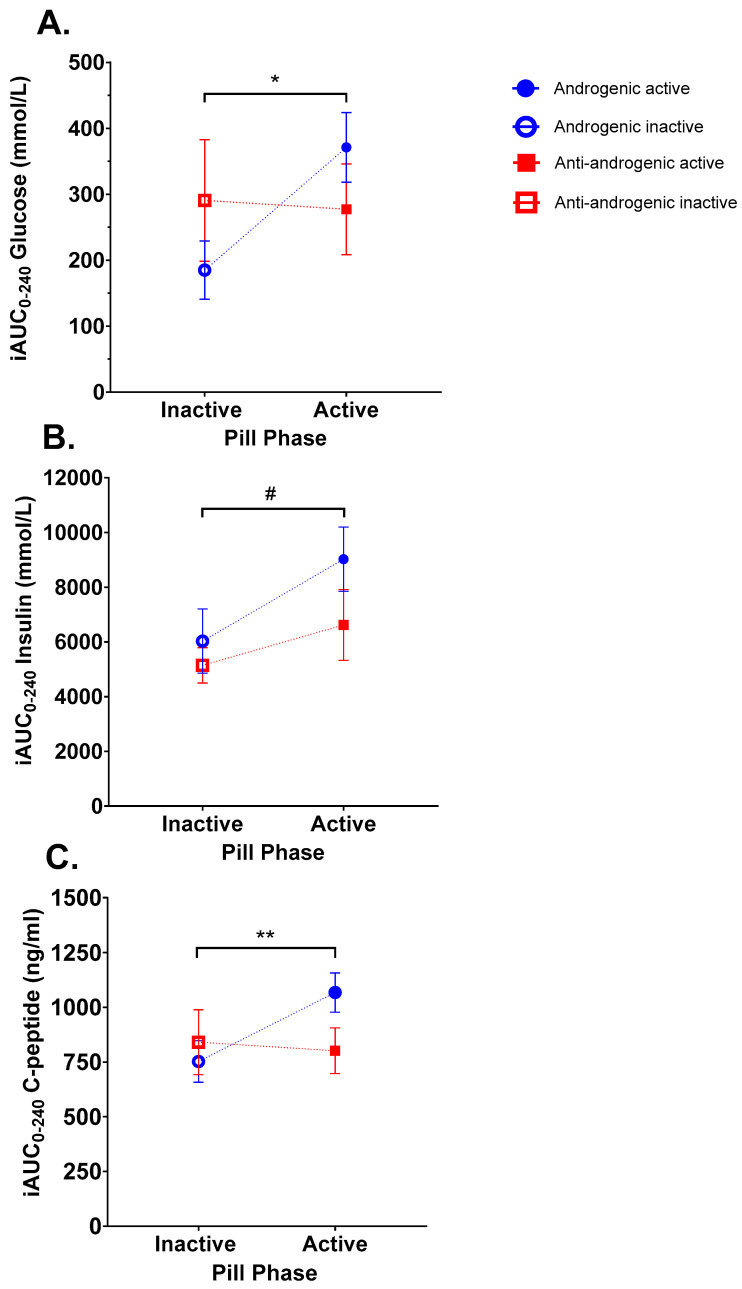
Net incremental area-under-curve (iAUC) from baseline to 240 min for plasma glucose (**A**), insulin (**B**) and C-peptide (**C**). Women using oral contraceptive pills (OCPs) containing androgenic progestogens (n = 13) during the active (blue closed circles) and inactive phases (blue open circles). Women using OCPs with anti-androgenic progestogens (n = 8) during the active (red closed squares) and inactive phases (red open squares). Significant interaction between pill type and phase observed for glucose (* *p* = 0.034) and C-peptide (** *p* = 0.005). Significant effect of pill phase observed for insulin (# *p* < 0.001). Mean ± SEM.

Figure 1 and Figure 2 show postprandial changes (relative to fasted baseline) in plasma glucose, insulin and C-peptide over 240 min and net iAUC for each, respectively. Plasma glucose concentrations reached a peak 30mins after glucose ingestion in both OCP groups and phases, decreasing thereafter (Figure 1A). A main effect due to pill phase (*p* = 0.002) but not pill type (*p* = 0.324) was detected by analysis of timecourse data (Figure 1A), along with a significant interaction between pill type and phase (*p* = 0.001). However, *post hoc* pairwise comparisons within each pill type found no statistical difference between phase at any individual postprandial time point. As seen in Figure 2A, comparison of net iAUC demonstrated no difference in iAUC glucose due to pill type or phase alone. However, an interaction between pill type and phase was observed (*p* = 0.034), with net iAUC higher in the active than the inactive phase in the androgenic users (*p* = 0.005), but no such difference was detected between phases in anti-androgenic users.

A main effect of pill phase (*p* < 0.001), but not pill type (*p* = 0.210), was detected in insulin timecourse data (Figure 1B). Time-to-peak insulin was shifted in the androgenic pill users from t = 30 (inactive) to t = 60 (active) postprandial with *post hoc* comparisons demonstrating higher insulin levels at t = 60 (*p* = 0.026) and t = 90 (*p* = 0.011) in the active phase for androgenic pill users than in their inactive phase, but there was no significant difference at any other timepoint, nor were there any such changes observed in anti-androgenic users. As seen in Figure 2B, the net iAUC for insulin also differed according to pill phase (main effect *p* = 0.001), with *post hoc* comparisons indicating higher iAUC in the active phase versus inactive in androgenic users (*p* < 0.001) but no difference between phases for anti-androgenic users.

Overall, there was a significant interaction between pill type and phase on postprandial response in plasma C-peptide across the 240 min measurement period (Figure 1C; *p* < 0.001), with *post hoc* testing showing values higher during the active phase versus the inactive phase in the androgenic users (*p* < 0.05), but there was no difference observed between phases for anti-androgenic users. C-peptide was significantly elevated in androgenic pill users during the active versus inactive phase at t = 90 (*p* < 0.01) but did not differ between phases at any other timepoint nor at any timepoint in anti-androgenic users. Similar differences were observed in C-peptide net iAUC (Figure 2C), with a significant interaction between pill type and phase (*p* = 0.005) and *post hoc* comparisons indicating a significantly higher iAUC during the active as compared to the inactive phase in the androgenic group (*p* < 0.005), but no such difference was observed in the anti-androgenic group.

## 4. Discussion

In the present study, in response to a 60 g oral glucose bolus, we observed an increase in mean iAUC of postprandial glucose, insulin and C-peptide of approximately 100%, 50% and 42%, respectively, in the active versus inactive phase of women taking OCPs containing androgenic progestogens, indicating that androgenic OCP formulations may impair glucose tolerance and increase insulin resistance. Similar trends in iAUC postprandial glucose and C-peptide have been observed by others but with a decrease in iAUC for insulin, indicating heightened hepatic insulin clearance [18]. In contrast, our results demonstrated a potential increase in insulin secretion (as indicated by increased postprandial C-peptide levels), but no such compensatory increase was seen in clearance–possibly resulting in the sustained elevation in circulating insulin observed across the 3 h postprandial period in the active phase of androgenic OCP users. Furthermore, the iAUC changes in glucose and insulin seen in our study in response to active androgenic OCP types are similar to those seen in women prescribed an androgenic (levonorgestrel and ethinylestradiol) OCP in a randomised controlled trial [19], when compared to their baseline, pre-OCP values. These authors also saw “negligible” change in these parameters in women prescribed an anti-androgenic pill type (nomegestrol acetate and 17β-oestradiol). However, it should be noted that the trial did not investigate the reversibility of such changes, measurements were compared across a relatively prolonged period (approximately 6 months) during which factors like a participant’s diet and lifestyle could have conceivably altered, and participants were not excluded based on prior or current hormonal contraceptive use, with a pre-treatment washout period of only one menstrual cycle. Indeed, previous studies in this field have either compared OCP users with a separate non-user control group or been conducted as an interventional study where non-users commencing OCP use are tested after some months, with differences and changes in confounding variables like diet and exercise across this time often unaccounted for. By comparison, the crossover study design used in the present study, where each woman acts as her own “control”, with a “wash-out” period to match the OCP manufacturer’s instructions, and all participants having completed the study within one pill cycle (i.e., 28 days), offers greater statistical power for studies with smaller numbers of participants and less confounding. Furthermore, recently published work [20] supports the timing of study days and blood sampling used in the present study, demonstrating differences in estradiol and progesterone levels between the two phases, and between the two pill types, studied here. It is however acknowledged that despite reducing inter-individual variability, this study is not without limitation and potential bias. In particular, (i) sample size and power: although *a priori* power calculation was undertaken, the small sample size of the present study, especially when divided into subgroups, may limit the generalizability of the findings; (ii) potential participant selection bias: participants were not randomly assigned their OCP type, and therefore, bias may have been introduced to the findings, despite explicitly excluding individuals with self-reported endocrine or hormone disorder (e.g., polycystic ovarian syndrome) from participation; and, (iii) *post hoc* modifications: as potential differences between androgenic and anti-androgenic pill types were only identified after preliminary analysis of the results, this *post hoc* modification of study design is an additional source of potential bias. In particular, there was no stratification for pill type in the randomisation plan, and therefore, the sequence of visits was not balanced between the two pill groups—although given both study visits occurred across a relatively short time period, and the nature of the physiological parameters (i.e., objective measures) assessed here, it is unlikely the sequence of visits would have had a large bearing on these results. As such, these results should be interpreted with caution, and fully controlled, randomised controlled trials are warranted to confirm the effects observed here.

The rising incidence of adverse metabolic health, including impaired glucose tolerance, is linked to an increased prevalence of cardiometabolic diseases in women, particularly those of child-bearing age [5,21]. Due to the large number of young women using the OCP worldwide, and the known influence of sex hormones (endogenous) on glucose metabolism [22,23], considerable need exists to investigate the extent to which exogenous sex hormones can contribute to the development of metabolic dysregulation. Evidence to support a link between type 2 diabetes and OCP use is beginning to accumulate. For example, in a cohort of >80,000 French women, extended exposure to endogenous female sex hormones, such as due to later menopause, was associated with reduced type 2 diabetes risk, whereas exogenous hormone exposure in the form of OCPs was associated with increased risk [24]. Similarly, compared to women who have never used OCPs, the prevalence of diabetes has been shown to be significantly higher in both post- and peri-menopausal women who had previously used OCPs [25,26]. Recently published findings of longitudinal cohort study (followed in Finland since birth in 1966) demonstrated a near 4-fold increase in the risk of developing prediabetes in these (now) perimenopausal women who had used OCPs for 10 years or more, compared with non-OCP users [27]. However, as discussed earlier, research in this area is lacking and largely cross-sectional. Importantly, the present study is the first crossover study to assess fasting and glucose-stimulated glycaemic profile in habitual OCP users during their active phase and inactive phase.

The risk of venous thromboembolism in OCP users was first acknowledged in 1966, and despite extensive re-formulation, the incidence of thromboembolism in OCP users is over twice that of non-users [28]. Specifically, lower levels of HDL-C and higher levels of triglycerides are associated with increased risk of thromboembolism in women [29,30]. Our results at baseline during the active pill phase reflect these adverse changes and agree with previous observations in users of similar OCP formulations [9,31]. Changes observed in lipid profile during the active phase, such as increased triglycerides, coupled with a decrease in HDL-C suggest that the OCP can increase cardiovascular disease risk [32]. Significant differences seen between pill types for HDL-C and total cholesterol/HDL-C ratio may indicate that anti-androgenic pill types may ameliorate this risk somewhat, although it is the estrogen component that is thought to elevate hepatic lipid synthesis, increasing triglyceride levels in a dose related manner [33].

Despite other studies having reported slightly differing results when considering glucose, insulin and C-peptide responses individually, the general consensus in the literature is that androgenic OCPs are detrimental to insulin sensitivity, although the exact mechanism remains unclear. Whilst the progestin component differs in these pill types compared to their anti-androgenic counterparts, it has been suggested that observed increases in pancreatic β-cell response and changes to insulin clearance may in fact be mediated by the estrogen component of the combined OCP [34,35,36]. Furthermore, this apparent synergistic effect of estrogen in the presence of progestin is supported by an apparent lack of effect of progestin only “mini-pills” on insulin sensitivity [37].

In combination with EE, differing progestins differentially affect insulin sensitivity [37] and blood lipid profile [38]. In our study, progestins structurally related to testosterone, such as levonorgestrel, desogestrel and norethisterone, exhibited differing effects on a range of metabolic and hormonal biomarkers as compared to anti-androgenic progestins such as cyproterone acetate (which is structurally related to progesterone) and dienogest (despite its structural link with testosterone) [39]. A meta-analysis [40] investigating the effect of oral contraceptives on lipid and carbohydrate metabolism has attempted to decipher the influence of the progestin component, with 80 of the 82 studies (97.6%) analysed containing EE as the estrogen component of the OCP. Although there was some variation in response between different OCPs, the authors drew similar conclusions to our study—namely that anti-androgenic progestogens dienogest and cyproterone acetate demonstrated the most favourable metabolic profile, with the most used progestin component of OCPs in our study, levonorgestrel, deemed to show the least favourable profile. A review of the metabolic actions of androgens [41] affirms the role of hyperandrogenic conditions such as PCOS and testosterone excess in glucose intolerance and insulin resistance, which has long been acknowledged and attributed to reduced glucose uptake by skeletal muscle. However, treatment with androgen receptor antagonists does not always improve insulin resistance; thus, androgens may not entirely be responsible for insulin resistance but rather a contributing factor. Other proposed mechanisms include excess androgen receptor activation in β-cells as an adverse determinant of β-cell function.

There is credible evidence that low levels of SHBG are also a key risk factor to the development of type 2 diabetes [42], with a 2015 meta-analysis [43] indicating the connection between SHBG and the adverse influence of excess testosterone on glucose metabolism. The later publication concluded that OCPs decreased total testosterone by a mean value of 31% in comparison to our study where a mean decrease of 38% was seen acutely in users of OCPs containing anti-androgenic progestogens. However, a decrease was not seen for OCPs containing androgenic progestogens—a result which is challenging to explain as the view of the meta-analysis [43] was that maximal effect of an OCP on testosterone can be observed after 3 weeks of “active pill” use. However, it was also concluded that free testosterone reduced by approximately 60% with OCP use [43], not only by suppression of androgen synthesis but due to an increase in SHBG. In comparison to the mean SHBG levels of <100 nmol/L previously observed in young females [31,44], women in the present study taking androgenic OCPs showed SHBG levels in the upper range observed in these studies (~133 nmol/L in the active phase), but SHBG levels in anti-androgenic users were far in excess of these limits (~335 nmol/L in the active phase). Thus, in our study, it appears testosterone levels are countered by high levels of SHBG, particularly in women using OCPs with anti-androgenic progestogens, thereby reducing free testosterone to an extent that the androgen-driven anticipated increase in insulin resistance was mitigated in that group relative to the androgenic pill users.

In addition to testosterone, other hormones are known to affect glucose metabolism, with their overall effects described as either anabolic (insulin) or catabolic (epinephrine, cortisol, glucagon, and GH) [45]. It is worth noting that while SHBG is often negatively correlated with circulating cortisol concentrations, the high plasma cortisol levels observed in the present study have long been recognized in association with OCP use [46], with levels more than doubling after 6 months of OCP, even with the introduction of more recent formulations [31]. Given the known risks of elevated cortisol to metabolic health [47], such an increase has the potential to contribute to an increased risk to glucose homeostasis over a prolonged period of OCP usage.

## 5. Conclusions

In summary, our research demonstrates a significantly amplified blood glucose, insulin and C-peptide response to an oral glucose bolus in the active versus inactive phase for women taking OCPs containing androgenic progestogens. While carefully controlled randomised controlled trials are required to confirm these findings, and the underlying mechanisms of this amplification remain to be elucidated, with the prevalent use of OCPs by women of child-bearing age, such effects merit more attention within the clinical setting. Investigations of response to a mixed meal and/or energy and macronutrient profile which is more representative of daily life are also needed. Such future research may assist in the development of personalised prescribing recommendations and/or dietary advice for OCP users who may be at risk of developing a cardiometabolic disease, including type 2 diabetes.

## Figures and Tables

**Table 1:** OCP usage by brand name and hormonal composition.

OCP Brand Name	No. of Participants	Estrogen (Dose µg)	Progestogen (Dose µg)	Progestin Class	Progestin Generation
Androgenic					
Ava 20	2	EE (20)	Levonorgestrel (100)	19-nortestosterone	2
Brevinor	1	EE (35)	Levonorgestrel (150)	19-nortestosterone	2
Levlen	7	EE (30)	Levonorgestrel (150)	19-nortestosterone	2
Marvellon	1	EE (30)	Desogestrel (150)	19-nortestosterone	3
Microgynon	1	EE (30)	Levonorgestrel (150)	19-nortestosterone	2
Norimin	1	EE (35)	Norethisterone (500)	19-nortestosterone	1
Anti-androgenic					
Ginet	7	EE (35)	Cyproterone Acetate (2000)	17α-hydroxyprogesterone	3
Jeanine	1	EE (30)	Dienogest (2000)	19-nortestosterone	4
Total (n)	21				

Abbreviations: EE, Ethinylestradiol; OCP, oral contraceptive pill.

**Table 2:** Participant characteristics.

Characteristic	All	Androgenic OCP Users	Anti-Androgenic OCP Users	*p*-Value
n	21	13	8	
Age (y)	24.4 ± 6.5	23.1 ± 6.0	26.6 ± 7.1	0.23
Weight (kg)	61.4 ± 5.0	60.6 ± 4.0	62.5 ± 6.5	0.41
Height (cm)	166 ± 4	166 ± 5	166 ± 4	1.00
BMI (kg/m^2^)	22.4 ±1.8	22.1 ± 1.7	22.8 ± 2.1	0.43
FFM (kg)	42.2 ±3.9	41.6 ± 3.8	43.3 ± 4.2	0.33
% Body fat	31.4 ± 5.2	31.2 ± 5.6	31.7 ± 4.9	0.84

Abbreviations: BMI, body mass index; FFM, fat-free mass; OCP, oral contraceptive pill. *p*-value provided for differences between androgenic OCP users and anti-androgenic OCP users tested by unpaired *t*-tests. Mean ± SD.

**Table 3:** Baseline (fasting) levels of glucose, hormonal and lipid blood biomarkers for inactive and active OCP phases and for androgenic (n = 13) and anti-androgenic OCP groups (n = 8).

Measure	Androgenic OCP Users		Anti-Androgenic OCP Users		*p*-Value		
	Inactive	Active	Inactive	Active	OCP Phase	OCP Type	Inter-Action
n	n = 13	n = 13	n = 8	n = 8			
Glucose (mmol/L)	4.91 ± 0.12	4.84 ± 0.10	4.76 ± 0.09	4.83 ± 0.07	0.979	0.573	0.416
Insulin (µIU/mL)	6.77 ± 1.27	6.99 ± 1.00	7.79 ± 1.63	7.73 ± 1.98	0.893	0.655	0.810
C-peptide (ng/mL)	1.88 ± 0.17	1.74 ± 0.11	1.79 ± 0.19	1.93 ± 0.21	0.542	0.981	0.021
HOMA1-IR	1.51 ± 0.30	1.53 ± 0.24	1.69 ± 0.38	1.66 ± 0.43	0.978	0.720	0.850
HOMA2-IR	1.36 ± 0.12	1.26 ± 0.09	1.29 ± 0.14	1.39 ± 0.15	0.531	0.999	0.019
Estradiol (pg/mL)	24.5 ± 4.6	11.0 ± 2.0	51.9 ± 13.7	14.3 ± 3.0	<0.001	0.020	0.081
Progesterone (ng/mL)	0.26 ± 0.04	0.26 ± 0.04	0.27 ± 0.04	0.31 ± 0.08	0.540	0.636	0.572
Testosterone (ng/mL)	0.25 ± 0.03	0.26 ± 0.04	0.38 ± 0.06	0.24 ± 0.05	0.021	0.351	0.008
SHBG (nmol/L)	117 ± 15	133 ± 20	266 ± 11	335 ± 25	<0.001	<0.001	0.003
Cortisol (nmol/L)	854 ± 63	985 ± 79	869 ± 122	1086 ± 119	0.006	0.630	0.456
HDL-C (mmol/L)	1.49 ± 0.08	1.34 ± 0.05	1.84 ± 0.15	1.77 ± 0.18	0.011	0.015	0.396
LDL-C (mmol/L)	3.30 ± 0.31	2.86 ± 0.30	2.51 ± 0.20	2.15 ± 0.20	<0.001	0.081	0.621
Triglycerides (mmol/L)	1.08 ± 0.07	1.21 ± 0.09	1.00 ± 0.10	1.17 ± 0.10	0.012	0.640	0.734
Total Cholesterol (mmol/L)	5.06 ± 0.29	4.61 ± 0.28	4.52 ± 0.21	4.20 ± 0.31	0.002	0.262	0.542
Total Cholesterol/HDL-C ratio	3.48 ± 0.25	3.50 ± 0.26	2.52 ± 0.15	2.44 ± 0.15	0.590	0.009	0.410

Abbreviations: HOMA1-IR, homeostatic model assessment for insulin resistance (original model); HOMA2-IR, homeostatic model assessment for insulin resistance (computer model); HDL-C, high density lipoprotein cholesterol; LDL-C, low density lipoprotein cholesterol; OCP, oral contraceptive pill; SHBG, sex hormone binding globulin. Mean ± SEM.

## Data Availability

The datasets generated during the current study are available from the corresponding author upon reasonable request. No applicable resources were generated or analysed during the current study.
